# Precision public health in the making: examining the becoming of the “social” in a Swiss environmental health population-based cohort

**DOI:** 10.3389/fsoc.2023.1219275

**Published:** 2023-12-15

**Authors:** Nolwenn Bühler

**Affiliations:** STSLab, Institute of Social Sciences, University of Lausanne, Lausanne, Switzerland

**Keywords:** precision public health, exposome, biomedicalization, reductionism, holism, environment, cohort

## Abstract

Expanding the concept of “precision” or “personalized” medicine, personalized health and precision public health designate the use of various kinds of data—genomic, other omics, clinical, or those produced by individuals themselves through self-tracking—to optimize health interventions benefiting the whole population. This paper draws on an ethnography of the implementation of a population-based environmental health cohort to shed light on the reconfigurations brought about by the “personalization” of public health in Switzerland. Combining human biomonitoring and molecular epidemiology, this cohort aims to advance the science of the exposome, a notion referring to the totality of exposures to which individuals are subjected over their lifecourse. Addressing the tension between holism and reductionism, this paper points to the important gap between the promissory horizon of the exposome and the realities of practices. Situations of reductionism are defined as moments of friction and negotiation between different rationales and values, exposing what makes the science of the exposome, including its material, economic, institutional, and methodological constraints, as well as its imaginaries and values. Rather than opposing holism and reductionism, I emphasize that they constitute two sides of the same coin, as they both pragmatically enable action and produce situated versions of the social. This empirical case shows how reductionism operates at the chemical, biological, and populational levels to produce public health scientific and social values. It thus contributes to contextualizing the pragmatic and strategic choices made by scientists, as well as the values they favor, in a research environment marked by the predominance of biomedicine over public health. It shows how the reductionism of the “social environment” was made for a better social integration of the cohort into the Swiss political and scientific landscape of public health. Bringing together actors involved in public health and questions of environmental exposures, this cohort can be interpreted as a biomedicalization of public health research, as well as an attempt to socialize it through the broad category of the exposome.

## 1 Introduction

“*We need to measure exposure throughout the lifecourse, and exposure does not just mean if I smoke or I do not smoke, it also means where I live, what’s my income, what are my social interactions, how do I feel, how polluted is it, what do I eat, can I do physical activity, do I have a bike path near my home, what is my mental health, what is my ability to resist stress at work, my financial room for maneuver? It’s all this that influences health and we are interested in all of this. It’s true that we have this holistic vision of the determinants of health, including socio-cultural determinants.*” Bright-eyed, this molecular epidemiologist is explaining to me what a framework of the exposome can bring to epidemiological research and public health. As she enthusiastically enumerates different kinds of “exposures” or “health determinants,” I can feel the promissory potential of the exposome’s holistic ambition. Not only to produce a more comprehensive understanding of what influences health over the lifecourse, but also to include what I, as an anthropologist, consider to be often overlooked: people’s socio-cultural living environments. This paper draws on an ethnography of the implementation of a population cohort aiming to study the exposome, to ask how this holistic understanding of the “social” is translated into research practice.

The concept of the exposome emerged in the field of molecular epidemiology two decades ago, in the aftermath of the Human Genome Project (HGP) (Wild, 2005). Building on the limits of genomic approaches, it promises to “complement the genome” by integrating environmental, or non-genetic exposures, to understand the complex etiology of chronic diseases and causal pathways leading to ill-health. Transferring approaches from life and health sciences—especially sequencing techniques, biomarkers research, and exposure science (Canali, 2020)—it aims to go beyond the current limits of epidemiology and toxicology (Giroux et al., 2021). Unlike genetic material, which is stable and transmitted vertically over generations, the exposome is apprehended as dynamic, context-dependent, and evolving over an individual’s lifetime. Three kinds of “environment” are distinguished in this field: the general external, the specific (individual) external, and the internal (Wild, 2012). The general external environment comprises health determinants as varied as the climate, socio-economic status, or urban surroundings. It has a systemic and global dimension, which makes it hardly modifiable, whereas the specific external one is more behavior-related and assumed to be modifiable by individuals (Sillé et al., 2020). In exposomics, the internal environment is supposed to reflect the imprinting of the external environment on an individual’s biology. Facing the challenges of making low-dose multiple chronic forms of exposure visible, this field promises to reveal how the global environment “out there” is actually “within” us (Washburn, 2013; Creager, 2018). In this way, it becomes a “biosocial trace” (Müller et al., 2017; Chiapperino and Panese, 2021) or a “mediator between the naturalized worlds of the genome and the social world of illness and inequality,” operating as an “open signifier, an object shaped by its relationship to the genome, which then gives the genome new life as significant to the social world of public health” (Whitmarsh, 2013, p. 490).

The exposome is part of a broader shift, called the postgenomic (Richardson and Stevens, 2015) or the biosocial turn (Meloni et al., 2018), observed in other fields of life, health, and population sciences. This includes, for example, epigenetics, microbiome research, and other studies investigating gene–environment interactions (Ackerman et al., 2016). Departing from gene-centrism, this field renews how body-environment relations are understood. Reconfiguring the knowledge of how the environment permeates human bodies, it gives rise to new technoscientific imaginaries of public health science and politics (Shostak, 2013). Notably, the prospect of a better understanding of environmental exposures generates new possibilities for interventions in public health. For example, “precision public health” (Khoury et al., 2016)—or, in Switzerland, “personalized health” (Meier-Abt, 2016)—aims to use technoscientific advances in sequencing techniques and data sciences to intervene at the population level and reinforce public health. Expanding the clinical and disease-oriented focus of genomic or precision medicine, their ambition is to “provid[e] the right intervention to the right population at the right time” (Khoury et al., 2016, p. 398), “to promote health, prevent diseases and reduce health disparities by focusing on modifiable morbidity and mortality” (Khoury et al., 2016, p. 398).

While it generates the hype of a new paradigm (Canali, 2020), the value of postgenomics for public health is also understood more critically. Reacting to the declaration that 2016 would be the “year of precision public health” by the head of the US Office of Genomics and Public Health and Center for Disease Control and Prevention (CDC), critical voices have pointed to the techno-optimistic hype surrounding big data and postgenomics, and recalled the fundamental differences between public health approaches—which focus on structural vulnerabilities and favor prevention and health promotion—and precision medicine—which focuses on diseases and prioritizes treatment for individual patients. They highlight the risk of conveying a biological-molecular understanding of vulnerabilities, molecularizing “complex social phenomena, reducing the social experiences that condition population-level variations in exposure to individual-level molecular-level differences” (Senier et al., 2017, p. 107), emphasizing especially the risks of promoting interventions targeting individuals, and diverting precious resources away from the wider public (Chowkwanyun et al., 2018).

The social sciences have also both welcomed these developments as an opportunity, and taken a critical stance. On the one hand, they see it as an acknowledgement that structural forces shaping health are directly linked to the genome, providing scientific “proof” that politics and socio-economics are embodied, and that “biologies” have always been “local” and “situated” (Landecker and Panofsky, 2013; Lock, 2018; Niewöhner and Lock, 2018; Gibbon and Pentecost, 2019). This environmental recognition also opens up new possibilities for innovative interdisciplinary engagements (Niewöhner, 2015; Canali and Leonelli, 2022). On the other hand, valorizing the permeability of the postgenomic body might obscure other forms of violence (Roberts, 2017), and the future-oriented dimension of datafying public health could be used to postpone action and avoid addressing the uncomfortable realities of the present (Hoeyer, 2019). More specifically, the exposomic approach is seen as promoting a technicist and individualized vision of health, leaving out critical social and political questioning about social inequalities (Guchet, 2019).

Pointing to the tension between the reductionism and holism at stake in exposomics, Giroux shows that the objective of finding biomarkers, even though they reflect the external environment, remains caught in a causalist and mechanistic model of health and disease (Giroux et al., 2021). In addition, whereas exposomics create interesting opportunities to recognize the impact of environment on health, the main focus remains on the biological component of embodiment (Krieger, 1999), rather than the social. Giroux shows how, in fact, exposomics renews the historical tension between molecular and social epidemiology (Giroux, 2023), focusing, respectively, on how the internal environment of individual bodies react to exposures (Rappaport, 2011), and on the external environment’s biomarkers of health, for example the allostatic load^1^—a biomarker of chronic stress exposure (Serviant-Fine et al., 2023) —and their role in chronic conditions’ causal pathways, or, in other words, on “the biology of inequalities in health” (Senier et al., 2017; Vineis et al., 2020). In addition, Louvel and Soulier’s (2022) review of literature on the social production of inequalities, using the concepts of “biological embedding” and “embodiment of social experiences,” shows the important different meanings of the “social” in both approaches. Furthermore, the extent to which these are reconcilable is still debated (Yates-Doerr, 2020). If the exposome has holistic ambitions and aims to capture complexity, it also conveys a specific “technoscientific” form of holism and not a humanistic or experiential one, leading to what can be called “holistic medicalization,” which assumes that “each person’s whole dynamic life process is defined in biomedical, technoscientific terms as controllable and underlain a regime of control in terms of monitoring, quantification, prediction, risk profiling, early diagnosis, therapy, prevention and optimization that is all-encompassing” (Vogt et al., 2016, p. 310). In this way, these debates revive long-standing social sciences critiques of reductionism, essentialism, biologism, determinism and individualization, which have been present in technoscience and medicine since the early 1970s (Zola, 1972; Conrad, 1975). These critical insights are crucial in unfolding the promissory regime of exposomics and not taking its goals and assumptions for granted.

Still, the double-edged dimension of these critiques sheds light on the limits of critical narratives that have “run out of steam” (Latour, 2004), on the need to refine historical and epistemic genealogies of the “revival” of the environment in life, biomedical, and population sciences, and to examine how biological and social entanglements are produced in practice. What does the holistic “social” of the environment become in exposomics? To what extent is its complexity molecularized or reduced? Through which processes does reductionism operate, and with what effects? The need for a stable single referent is needed, for example, in mash-up studies bringing together data from different kinds and sources (Leonelli and Tempini, 2021). In other words, some situated forms of reductionism—or shrinking (Stengers and Isabelle, 2021)—are needed to grasp environmental exposures (Leonelli and Tempini, 2021). It is thus relevant to observe how, as data travel across levels, disciplinary cultures, and infrastructures, they retain some aspects of the social contexts they stem from Bauer (2008). Reductionism is not only produced through epistemic assumptions, scientific tools, and methods, but also through the politics and economics of scientific research. Accounting for how reductionism is made in practice, Pinel sheds light on the constraining logics of the entrepreneurial university, characterized by a market-driven institutional environment favoring a type of epigenetic research which is individualized and clinically centered (Pinel, 2022). In a similar way, Ackerman et al. (2016) show how the politics and moral economy of quantification lead to a shrinking of the environment in the interests of data standardization and harmonization. They especially highlight the pragmatic dimension of the scientists’ choices, as they are “compelled to make trade-offs or exchanges between competing priorities and commitments” (Ackerman et al., 2016, p. 197) in the name of objectivity, to make epidemiology more robust scientifically. Penkler et al. also soundly document the discomfort of Developmental Origins of Health and Disease (DOHaD) researchers, who attempt to capture how environmental factors such as deprivation, nutrition, and stress shape individual and population health over the lifecourse (Penkler et al., 2022). While scientists are eager to develop more complex understandings of the environment in their daily practices, they are confronted with established methodological tools, disciplinary infrastructures, budgetary constraints and institutional contexts that favor a reductionist understanding of the environment and individualistic approaches toward health. Reconstructing the pragmatic decisions at stake in the production of knowledge, these authors shed light on the multiple trade-offs scientists face, which lead them to focus on particular factors and easily-accessible data, to produce results that are aligned with the academic publication market, and the need to secure third party funding. It is important to recall, though, that social scientists also reduce complexities and wide, comprehensive sets of data to render them graspable through enmeshments in greater narratives—which also involves selection processes and adopting certain writing styles (Clifford and Marcus, 2010).

Drawing on these insights, in this paper I discuss some of the processes through which the complexity of the social is reduced, and how this reductionism is made to achieve better social integration—in the sense of the recognition, legitimacy and interest of the multiple publics of the environmental public health cohort—in the scientific and policy landscape of Switzerland. More specifically, I explore the becoming of the social in building a population study, a cohort, adopting an exposomic conceptual framework to study the impact of environment on health in Switzerland. Like other postgenomic projects, this health study can be apprehended as a “biopolitical assemblage where samples, data, and techniques from different contexts are temporarily brought together in particular configurations” (Bauer, 2008, p. 418). My objective is to account for the gap I have discerned between the promissory potential of the exposome as illustrated in the quote in the introduction, and the shrinking of holism and complexity I have observed over time. I adopt an empirically informed stance to document how reductionism operates in practices, and what is produced through the different forms it takes. I focus on a specific phase of scientific research—implementing the pilot phase of this cohort. This comprised building the infrastructure for the cohort, that is, setting up a database and a biobank, as well as the data flows necessary for their connection, testing the various procedures, instruments, and work instructions, as well as producing preliminary biomonitoring and epidemiological results. Over the course of its implementation, the cohort’s initial design was constantly reworked and negotiated. Thus, the implementation phase provides a relevant site in which to observe how the biological and the social entangle in precision public health “in the making.” I will first describe the cohort’s origin and the different versions of the environment that brought together the actors involved in this study. I will then analyze three situations of reductionism that I observed. Ultimately, this paper contributes to contextualizing the pragmatic and strategic choices made by scientists, as well as the values (Dussauge et al., 2015) they favor, in a research environment marked by the predominance of biomedicine over public health, to show how reductionism was used to promote the cohort’s social integration into the Swiss political and scientific landscape of public health.

## 2 Methods

This paper is based on a research project funded by the Swiss National Science Foundation in the framework of a Sinergia project: “Development of Personalized Health in Switzerland: Social Sciences Perspectives” (University of Lausanne, Institute of Social Sciences). My socio-anthropological sub-project adopted an empirical stance to explore environment-health relations and the making of “permeable bodies,” and to analyze the reconfigurations of public health research when it turns to “precision” or “personalized” approaches. I conducted an ethnography of implementing the pilot phase of a longitudinal population-based cohort which aimed to study the impact of environment on health. I made regular observations over the course of 4 years (2018–2022) by attending operational meetings,^2^ studying health examinations, visiting the biobank, and going to related events and conferences. In addition to ethnographic observations and informal discussions throughout the project, I conducted individual semi-structured interviews with members of: (a) the research team (5 from IT and biobanking, 6 with a public health scientific background, 3 with nursing knowledge, and 4 with public health policy expertise, *n* = 18); (b) external public health and biomedical experts and stakeholders (*n* = 3); and (c) cohort participants (*n* = 14). I collated a corpus of: (a) scientific and medical articles on public health genomics, human biomonitoring, exposomics, toxicogenomics, personalized health and public health; and (b) media articles referring to the environmental population cohort under study. Having adopted an engaged anthropology position, I also established a collaboration with the research team to develop a participatory approach. For this, we organized seven online focus groups reuniting 37 cohort participants (Bühler et al., 2023). In this paper, I draw mainly on my observations and interviews with the research team.

## 3 The “environmental” origins of the cohort

In 2008, concerned by the lack of knowledge about the impact of chemical exposures on health, a Green Liberal parliamentarian submitted a postulate asking the Swiss Federal Council to develop an assessment tool (Moser 08.3223), which was followed by similar postulates (FOPH, 2023). The Federal Council agreed to address this proposal and mandated an evaluation of existing biomonitoring data and projects. After identifying the important gaps that persist in the country concerning chemical assessment, the pilot phase of a human biomonitoring cohort was launched. This was in line with the national Health Strategy 2020’s declaration that it is important to use biomonitoring to improve the quality of life, and with the government’s legal requirements to regulate chemical products and surveil the population’s health. Molecular epidemiologists joined the project, aiming to advance exposome science by expanding the scope of biomonitoring to enable the collection of large sets of health data and biological samples. Through developing a prospective, longitudinal population-based health cohort, the initial focus on chemical exposure was extended to the broad domain of health, and renamed from a biomonitoring study to a health study. This setting brought together several groups of actors: (a) molecular epidemiologists, public health physicians and exposure scientists working in public health academic institutions and conducting research; (b) public health officers in charge of regulating chemical products; (c) IT and biobanking experts responsible for developing the infrastructure necessary to manage biological samples and health-related data; and (d) citizens, especially those selected as cohort participants. I refer to cohort participants as *cohortees*, and to the team of experts implementing the cohort, as *cohorters*.

The pilot phase of this health study ran from 2017 to 2022, produced a report that was approved by the Federal Council in June 2023, and showed the feasibility of a general population cohort at the national level. Two public health centers were involved, one in the German-speaking part and the other in the French-speaking part of Switzerland. Both aimed to recruit 500 residents, aged between 20 and 69, from their respective cantons^3^. The cohortees were randomly selected by the Federal Office of Statistics and received an official letter from the Federal Office of Public Health asking them to participate. They could then provide consent, and access several questionnaires to fill in on an online platform. Once those were completed, they were invited to a clinical research center where they underwent several health examinations, answered additional questionnaires on exposure and health status, and had anthropometric measures taken. During the visit, blood and urine samples were also collected. Some biosamples were analyzed directly, whereas others were prepared for biobanking and then sent to the cohort’s central biobank. The chemical products analyzed in the study were heavy metals, glyphosate, and Per-and polyfluorinated Substances (PFAS). Cohortees were also asked if they would like to use an app to record their meals for 2 weeks and wear a portable device—an accelerometer—to record data about their physical activity.

The impact of environment on health was a common concern that brought these actors to work together to develop the pilot cohort. Environment was a concern for some citizens,^4^ who worry about the health impact of living in a polluted, industrialized world, and the extent to which chemicals permeate their bodies and affect them in negative ways. Environment was a concern for public health representatives who are legally responsible for protecting the population’s health from chemicals. The pilot evaluation report showed that Switzerland lacks the evidence needed for an efficient state apparatus that regulates chemical products and assesses their risks. Environment was also a concern for molecular epidemiologists wanting to advance exposomic research. The prospect of building a longitudinal population-based cohort to investigate a great variety of forms of exposure in the general population, related to chemicals, but also to the built environment, nutrition, lifestyle and quality of life, provided a much-needed opportunity not only to evaluate the level of exposure (as in human biomonitoring), but also to “open the black box of the body” and understand how the environment affects health over the long term. Different realities of the environment relating to policy, science, and society thus constituted a common matter of concern, bringing together the actors implementing the cohort into a setting that was valued as suitable for responding to their needs and expectations. In addition, in the exposome’s conceptual framework, the environment can be understood as the external environment—social and individual—and the internal one—biological—all three being entangled. In the next sections I look at three situations of reductionism encountered in this cohort, which each represent different versions of the “social” environment and the way it relates to its biological counterpart.

## 4 Results

### 4.1 The substances of chemical exposures

A dozen people sit in a room, in the facilities of the Federal Office of Public Health (FOPH), looking at the slides projected on a screen. The room is small and I take notes on my lap as there is not enough space for us all to sit around the table. The goal of today’s operational meeting is to decide what kinds of chemical substances will be included in the study’s design. One of the team’s junior scientists, who has done some research to evaluate the costs and feasibility of chemical analyses, presents her results. I am impressed by the long list of substances appearing on the Excel spreadsheet and feel a kind of excitement at the idea that the study would account for the complexity of multiple forms of chemical exposures, if they were all included. However, it quickly becomes clear in the discussion that drastic choices will need to be made. The budget is tight, tubes are expensive, analyses are expensive, lab work is expensive. As the discussion goes on, the initial list shrinks more and more, until it finally includes only a little selection of substances. The discussion ends with the decision to get more information and discuss directly with the lab the price of the tubes and analyses.

This discussion illustrates how the complexity of the exposome and its ambition to capture a broad range of different exposures over time was reduced in respect to the number of chemicals studied. How did this reduction operate and how was this preliminary selection of chemicals made? Should substances be selected for their scientific interest and potential scientific value? To fill the knowledge gaps encountered by policy-makers in the context of risk regulation? Or to respond to the concerns of the citizens who alerted the government to their responsibilities in the first place? Scientific, policy, or social values were entangled in this situation of selecting which substances to analyze, but reducing their number meant that actors had to prioritize some over others. What emerged first in this situation was the evident financial gap between the cohort’s limited means and the price of the analyses needed to advance exposomic science. To reach the ideal of the exposome goal, a large amount of data and biological samples is needed. Quantity, including a very large sample size, is required to detect significant small differences and determine causal relations of ill-health, especially as exposures are chronic and low. The same is valid if one aims to understand how multiple forms of exposure interact and possibly potentialize each other, in what is called the cocktail effect. For this, a long-term approach is necessary, as a senior molecular epidemiologist explained:

*The distribution of chemicals is one thing, but many chemical effects are not understood, so you need to follow up on these people, especially mixture effects like low dose interactions between chemicals, for that you need to cohort with biobanks (molecular epidemiologist)*.

However, high quality data and samples are also needed to capture the complexity of chronic low-dose exposure to multiple forms of chemicals, as one of the cohorters expressed:

*Given that we are exposed to multiple substances, and that the effects are sometimes very weak, and sometimes unknown also, we need a great number of top quality samples to be sure that the variations we observe are not due to another factor (public health officer)*.

Omics analyses, such as metabolomics and proteomics, are highly sensitive to their immediate environment, so great care must be taken to maintain the quality of each sample. Quality refers here to controlling the parameters which may impact the samples, and tracking the samples from the health examination room where blood is drawn to the centralized biobank in another city where they are stored, including their passage via a preanalytical lab where plasma is separated through centrifugation and the blood samples are aliquoted. When debating which substances to include, the materiality of the blood or urine tubes was also discussed. Depending on the tube’s materiality, the biological substance within—e.g., blood—can be contaminated, rendering it difficult to determine whether exposure has come from the tube or from the external environment, thus possibly biasing the analyses’ results and rendering the cohorters’ work worthless.

Financial constraints and limited resources mean that some pragmatic and strategic choices need to be made. Trade-off situations such as the one described above are commonplace in scientific research practices. It is part of scientific work to have to adjust a project’s ambitions of what should or could ideally be done from a scientific perspective to match realities on the ground, such as financial limits and the materialities at stake. But, beyond this ordinary aspect, the responses given, the choices made, and the form of these trade-offs can teach us a lot about how reductionism operates, and the different enacted values attached to the versions of the “social.” In this case, when confronted with the choice of prioritizing some substances over others, the cohorters decided to select those which are of concern to the population: glyphosate for example, a chemical present in pesticides whose effects on health in Switzerland, as in other countries, are highly controversial (Adams, 2023); but also mercury, which is a chemical of concern for both the population and the regulators, since an industrial leak occurred in the canton of Valais, and there is a lack of threshold exposure values in the Swiss population (Parvex, 2014; Lambiel, 2017). Public health value was thus prioritized over the scientific one, in the sense of doing analyses which are relevant in the Swiss context, rather than favoring analyses which have great potential to be published in high-ranked academic journals. The value of public health was also visible in that the cohorters were responding to the population’s demand. By deciding to select substances that are debated socially and for which there is a high demand for scientific evidence, the cohorters’ objective was not only to meet the legal obligations for chemical regulation, but also to increase the interest of the population, who were envisioned as potential participants and beneficiaries of environmental health policies.

Several practical strategies were adopted to balance the need for high quantity and quality within financial limits. First, most omics analyses were postponed for later. This postponement strategy enabled the cohorters to reach maximal quality from a scientific point of view, within their financial limits in the present, as a guarantee of good analyses in the future, when other sources of funding might be available, or other teams and other projects could take over. Rather than cumulating the quantity of substances to analyze, they prioritized the quality necessary for exposomic analyses. The exposome’s holistic ambitions and chemical complexity were thus reduced for the sake of future scientific value. Another strategy consisted of making alliances with other teams which were interested in a specific substance, and could fund the analyses. “*We know more about water and soils than about human health in this country.*” This remark, heard several times during my fieldwork, expressed cohorters’ frustration concerning the lack of a large and comprehensive database about many kinds of exposure, and reflected the siloed distribution of monitoring responsibilities among the federal offices—one in charge of agriculture and food control, another in charge of surveilling water and soil, and a third in charge of chemical products used by humans. This institutional fragmentation of evaluating the presence of chemicals in humans, consumer products, and the environment is at odds with the comprehensive goal of the exposome and points to one of the institutional constraints the team met. To mediate this fragmentation, but also to increase the number of substances included in the analyses despite the cost, they established collaboration with several other federal offices and sub-projects focusing on more specific questions, for example, relating to nutrition and the substance of cadmium. Therefore, substances were added depending on the contextual interest and ability to fund analyses by parallel teams, with which specific agreements were made.

Looking at how reductionism operates indicates two important elements. First, it reveals the lack of public investment and the difficulties of obtaining sufficient funding for public health, especially cohort studies, in a country where there is no centralized database of exposures: data which could potentially be included to advance exposome science. In total, 68 million Swiss Francs (CHF) of public money were invested in the Swiss Personalized Health Network (SPHN)—launched in 2016 to develop the infrastructure necessary to enable the nationwide use and secure exchange of health data for research. For the cohort discussed in this paper, funding came from various sources, added over time, eventually reaching a total of about 3 million CHF (plus in-kind contributions from the scientific institutions) which, in comparison, shows lower public investment. While cohorters embraced the development of the pilot cohort as a way of advancing population-based research and environmental public health, in contrast with hospital-based molecular-focused projects such as those funded by the SPHN, the difficulties and reductionist choices they had to make reveals the challenge of reconciling the technoscience of the exposome with national goals to protect the population’s health in a country characterized by a historical weakness in this domain^5^ (Monod, 2022; Thieme et al., 2022). The financial difficulties encountered over the course of the project, balanced with efforts to increase its social integration, uncover in the background the high financial and human costs of implementing such a longitudinal population-based study, which are at odds with the lack of government investment in public health research.

### 4.2 The biology of social exposures

*The biggest user of Roundup*^6^ *in Switzerland is the national railroad company, because they weed their tracks with it. So if, all of a sudden, we could show that in Switzerland this makes a difference … also we are going to ask questions about mobility. If we realize that there’s a link between people who take the train and the quantity of Roundup, I can imagine that a fairly easy public health measure would be to say, well, stop using Roundup to weed train tracks, you've got to change, you've got to switch, no matter what the Monsanto lobby says, change now!*^7^

*Interviewer: You mean measures that wouldn’t target individuals as such, but that would make it possible to act to improve the population’s health by intervening on the national railroads?*

*Yes, a structural measure! I'm very fond of structural measures because they're the simplest. For individuals it's the simplest, well here we’ve put fluoride in your water and it protects you from cavities and you're not even aware of it and you don't even have to say, “ah those public health doctors who prevent me from doing this or that”… So I like structural measures, you're starting to know me. To improve walkability in the city or certain neighborhoods, we could imagine something very simple. If we realize that in more or less disadvantaged neighborhoods we find that people do less physical activity because they don't have the time, because in terms of health knowledge, it's not enough, well we could imagine putting in place urban planning or measures like that to improve physical activity without them even realizing it! (molecular epidemiologist)*.

The enthusiasm of this molecular epidemiologist was contagious, and I remember being hooked by the promise of a science that would eventually reinforce so-called structural public health measures, intervening in the environmental sources of ill-health, rather than targeting individuals and making them responsible for their own wellbeing. In this cohorter’s narrative, these measures were associated with simplicity, freedom, and a lack of the moral judgment that often accompanies interventions aimed at individual behaviors. Instead of blaming or stigmatizing people for their lack of physical activity, the epidemiologist preferred measures targeting their living environment. Nevertheless, the ideal of structural measures defended here appeared to me at odds with the great expectations for biomarkers this scientist expressed later in the interview. The cohort’s potential to identify biomarkers, and the ability to improve scientific understanding of how the classical social determinants of health might cause ill-health, by “un-black boxing” the body to evaluate and improve public health interventions was palpable in many of the discussions I had with cohorters. Their hope that the ‘social determinants’ credibility and legitimacy would be strengthened, if they could prove the biological impact of social exposures by identifying biomarkers, was especially striking, as the following quote illustrates:

*If we can show that being poor for forty years leads to different biomarker profiles, like cytokines levels—because we have this tendency to only believe biological facts—it will help people to understand that poverty is not some esoteric concept, that it has a biological correlate (molecular epidemiologist)*.

This quote illuminates how public health scientists invest in exposomic science because of the biologization or molecularization of the social it brings. The idea of finding biomarkers, or biological signatures of the social which inform about the molecular pathways leading to chronic conditions, is invested with much promissory potential concerning the possible public health measures deriving from such a study. Molecular reductionism in this sense is searched for, as a condition of the possibility to be taken seriously by health authorities and to make a difference in public health interventions. The exposomic ideal is not only about understanding the causal molecular pathways of disease, but also about finding actionable knowledge to address or prevent it. Finding causes is deeply entangled with the possibility of interventions. However, the molecular signature of the social and the possibility of intervening structurally become almost interchangeable in cohorters’ discourses around biological reductionism, a strategic passage point, as the following quote illustrates:

*I think the biggest opportunity of these biomarkers and, by data, I mean all mixed markers and imagery markers, is that we can investigate mechanisms between lifestyle and environment to disease development so we can look into the biology on the pathway from these risks to a health effect, and we know that one of the important factors of causal understanding is understanding biology. To me, it is the biggest tool of preventive research and that’s what exposome research is about (molecular epidemiologist)*.

These quotes illustrate well the predominance of a biological regime of proof. A molecular understanding of ill-health is envisioned as being both the most solid scientifically, able to produce more robust evidence of the impact of social determinants of health, but also as being more actionable, as biomarkers might be used to develop evidence-based public health interventions.

What is the social correlate of these biological traces? How are poverty, living environment, walkability or use of public transport translated into the scientific idiom of the cohort study? Discussions about which variables and questionnaires to include provide another situation of reductionism. The exposome’s holistic ambitions were reduced, not only through the predominance of biology’s capacity to translate the social determinants of health into biomarkers, but also in the social elements which were covered. In the cohort, two questionnaires aimed to document different situations of exposure, and included the channels through which chemicals might permeate bodies. In addition, there were questionnaires on participants’ quality of life, nutrition, medical history, and general state of health. Using an app to record their physical activity and photograph meals was also proposed to cohortees. Due to the biomonitoring origin of the cohort in regard to the FOPH’s legal mission to monitor exposure, and its focus on the chemical environment, exposure questionnaires were more detailed than those on socio-economic health determinants. However, the idea was to be able to characterize cohortees’ socio-economic backgrounds by recording their history of occupational activities and the nutrition questionnaire, and possibly to understand how far these were related to their health.

Reductionism operated in different ways here. Exposure was understood as contact with a substance and the identification of the parts of the body, or activity that could lead to it. To determine targetable causes, there was a need to offer distinct variables that cohortees could select in questionnaires. Situations of exposure were predefined and some specific practices used as a proxy for how much a person was exposed to a substance: for example, how many times in the last few days someone may have held a grocery store receipt, which might contain the endocrine disruptor bisphenol, or how often they encountered difficulties making ends meet, as a proxy of precariousness. In addition, for data to be recognized scientifically and be comparable with other similar studies or combined with other datasets to possibly increase the quantity of data in the longer term, the questionnaires needed to be validated. This meant that they had already been developed and validated in other studies. Thus, the need for scientific standardization limited the possibility of asking more detailed questions about multiple and dynamic forms of exposure, how those might change over time and how they are situated in a broader socio-cultural context and in interactions. Thus, standardization and interoperability informed the prospective design chosen. It implied that the goal was to collect as much data and samples as possible, with the idea that, in the longer term, the cohort could be scaled up to the whole Swiss population, and datasets and biobanks used in comparison or in combination with others. This reductionism operating in the name of future science and interventions clearly illustrates the methodological challenge of shifting from epidemiology to an exposomic data-driven approach, and the ways that harmonization and standardization principles at the core of personalized health initiatives shape the becoming of the social in such a cohort. While the social context of exposure, as well as social health determinants such as poverty are in a way reduced through their datafication, the greater value granted to biomarkers due to their ability to be used in interventions can be read as a means to make public health better recognized and more robust scientifically. Moreover, favoring standardization is also a condition of increasing the possibility for data and samples to circulate and gain increased scientific value in other studies. By not focusing on a specific disease or hypothesis, and taking a prospective approach to health which aims to collect as much data as possible, the goal is to keep the possibility of discovering key health determinants or biomarkers open. It is thus regarded as a way of reinforcing scientific prevention and health promotion in the future.

### 4.3 Recruitment and the stratification of exposures

The need to have Swiss data about environmental exposures was one of the main reasons given to justify setting up the cohort. Exposure was thought of as being mediated locally, and the goal was to identify groups of the population who might be more at risk, to develop public health strategies that are more stratified, in the sense of differentiated and sensitive to the geographical and socio-cultural context of individuals’ lives, as the following quotes illustrate:

*If you live in Valais where la Lonza dumped mercury into the "Grossgrundkanal" for 40 years, if you grow your carrots there it's not the same as if you grow them in Schaffhausen. The exposure of people in Geneva is not the same as that of people in Grisons (public health officer)*.

*We might end up with usual lifestyle recommendations, however, what’s new is the innovative potential to characterize the situation in Switzerland, and identify groups of the population—geographical areas, age categories, things like that—which are much more at risk than previously thought, or which are under the radar because, in the end, we don't have much data on the whole population, on what happens at the neighborhood level (molecular epidemiologist)*.

To this end, a prospective design was chosen. People residing in Switzerland, aged between 20 and 69, were randomly selected by the Federal Office of Statistics and then invited by letter to log in online, where they could give their consent and start filling in questionnaires. There was no specific stratification of the population in advance; the idea was that the random selection ensured that the cohort was representative of the population in the pilot cantons. The randomly selected cohort works as a reduction tool that enables researchers to translate the diversity of the general population’s characteristics and specificities. In addition, this recruitment method is considered to be the “gold standard” of epidemiology, as the best design to maximize external validity and thus the findings’ generalizability:

*Prospective design is ideal [from a scientific perspective] because it allows us to avoid the risk that our results may be biased. When you do a retrospective study, you have a lot of selection bias: maybe some of the people you could have taken on as controls are dead, so you are not really comparing everyone if they are not there. But if it is prospective, you choose people randomly from the start, and you follow them over time, then it is potentially representative (molecular epidemiologist)*.

To be able to differentiate significative differences between the overall population and “at risk” groups, a great quantity of data must be collected. The more precision that is wanted, in the sense of local and specific, the larger the cohort population needs to be. But it is challenging to recruit and retain people to cohort studies over a long time (Marques et al., 2020). Recruitment and participation rates therefore constituted a major concern for cohorters. During the operational meetings I attended, the choice of the best recruitment strategy was discussed several times. Alternative ways of recruitment, such as mobilizing general practitioners or distributing flyers in pharmacies were debated for example, in the hope that this might increase participation. This would have involved collaborating with healthcare professionals in frontline contact with the population. In an informal discussion with cohorters, the idea that anyone residing in Switzerland could participate if willing, thereby transforming the whole Swiss population into a data reservoir, was also imagined. In all scenarios, logics of exclusion and inclusion were present. Some in the team were more in favor of opening up possibilities of recruitment to increase participation rates—enough quantity to enable a significant stratification of results—while others maintained the importance of random selection as the most robust scientific approach. Randomly selecting the cohortees was preferred for scientific reasons, but a convenience sample based on distributing flyers in strategic places and informing people by word-of-mouth was also tested in a subgroup of the cohort: the vegan and vegetarian individuals. The random selection of a population sample was considered a better option in the sense that it allowed more control for the scientists involved, by enabling them to monitor the recruitment parameters: who was contacted, who gave informed consent, who dropped out. In contrast, relying on word-of-mouth and recruiting people based on their affinities and concerns would reduce cohorters’ control, thereby becoming weaker according to scientific criteria. In the latter case, the loss of control might be remediated by the quantity of data, which is more aligned with data-driven approaches. However, opting for scientific robustness by controlling the cohort parameters also related to the prospect of producing better publications based on the results, and thus a better scientific valorization of the work performed in the pilot phase of the study.

To address the challenge of participation, specific efforts were made from early on to understand cohortees’ motivations, expectations, and obstacles to participation, in the belief that understanding them could facilitate and increase their participation in the long term. Cohorters tried to balance their scientific need for a large quantity of data with the amount of clinical labor (Mitchell and Waldby, 2010) that could be expected from cohortees:

*The difficulty is measurement error. None of the tools we have available is perfect. There are measurement errors in all the tools. The challenge is how to do this measurement without it being too much of a burden? We can't send out a 300-question questionnaire to people every week. I mean, after a while, they just get bored. Then there's the photo app [for food], it is interesting to test other models, but taking photos of everything you eat, it lasts a few days but not much more, because you eat every day! You eat several times a day! So how do you find the right balance between capturing the right exposure, measuring it properly, and not, how can I put it… exhaust people? (molecular epidemiologist)*.

*Of course, we can't collect everything either, and it's not our goal to have people wearing sensors 24 hours a day or whatever, but the idea is to have a panel of data that will allow you to start something (public health officer)*.

To address this challenge, they developed a questionnaire on attitudes toward research and willingness to participate, and implemented a public involvement initiative with several focus groups. While this was done to increase participation, and thus data collection, it also revealed how cohorters felt about the social integration of the cohort in the population. At the end of the pilot study, the overall participation rates reached 14% (Bourqui et al., 2023), which is similar to those of other cohort studies (Kuss et al., 2022). It also became clear that those participants who were possibly more exposed, due to their poorer socio-economic living environment, were less included and harder to reach through the means of recruitment used in the cohort. Instead of “precision” in the stratification of exposure, the results were valorized as representing the “normality” of the general population, constituting a good basis for comparisons with more at-risk groups, which could be targeted later.

The discussions and choices made around recruitment strategies can be interpreted as another situated form of reductionism of the social, as only a small part of the population was initially selected, and tendentially more socio-economically privileged, more feminine and older people (Bourqui et al., 2023) actually participated, so constitute the representative sample of the population. While attempting to shift epidemiology toward big data and exposomics, this example illustrates how the standard of random selection constraints informed and oriented the trade-offs made by the cohorters. If the results far from provide a biological signature of how poverty shapes environmental exposures, as the cohorters had first envisioned when talking about the exposome, the choices made also reveal what they care for most: the cohort itself and building the infrastructure. In this sense, this reductionism is productive, as it reveals how the social valorization and the scientific value of the cohort combined to pragmatically pave the way to advance such public health cohort studies in Switzerland.

## 5 Discussion and concluding remarks

In the social sciences of medicine and health, reductionism has been used to criticize the biologization, molecularization, or genetization of complex social phenomenon, such as the embodied experience of ill-health and the multiple enactments of health, illness and healing processes. The complexity of how power, economics, the living environment, interactions, institutions, biomedical knowledge, technologies, or metrics shape health and permeate bodies over the lifecourse gets lost in translating the social into a causalist mechanistic model of health (Yates-Doerr, 2020). Postgenomic approaches renew the understanding of biosocial entanglements, allowing revisiting the tension between holism and reductionism. Do they represent new forms of holistic medicalization, of technoscientific holism (Vogt et al., 2016), or do they open up possibilities for renewing interdisciplinary dialogue and co-laboration (Landecker and Panofsky, 2013; Niewöhner, 2015)? In this paper, I have addressed this question through an ethnographic exploration of an exposomic Swiss cohort in the making. I have approached reductionism as enacted in practice, to understand how it operates in the domain of the exposome, which aims to capture the complexity of multiple exposures throughout the lifecourse, and to bring together technoscientific advancements with public health objectives and agendas.

Examining the tension between holism and reductionism, this analysis has pointed to the important gap between the exposome’s promissory horizon adopted by the cohorters in my fieldwork and the realities of research practice. While the exposome is a notion conveying a holistic and comprehensive understanding of the health determinants and forms of exposure affecting individuals over their lifetime, in the realities of research in the field, different forms of reductionism of the social environment can be observed at the: (1) chemical, (2) biological, and (3) population level. In each situation pragmatic compromises and strategic choices needed to be made, and some values emerged as more important than others. In the first situation described above, the limited number of chemicals chosen illustrates the lack of funding for precision public health research, as well as the prioritization of samples’ scientific quality and the public health value of the substances analyzed. The second situation illustrates the predominance of the biological signature of the social environment used to develop evidence-based public health interventions, as well as the power of standardization and harmonization imperatives in reducing the social milieu, at stake when epidemiology shifts toward exposomic science. The strategic choices made in the present aim to reinforce the scientific value of the cohort, to be scaled up in the future. Finally, the third situation analyzed shows well the difficulties of reconciling epidemiological methods of constituting a “population” with the data-driven, more open-ended approach of the exposome. Debates about recruitment strategies reveal a tension between prioritizing the *quantity* of data and sample collections, and their *quality*, enabled by optimum control over certain parameters of the cohort’s population. While it illustrates random selection’s limitations in representing population diversity, and reflects the social stratification of exposures, it also sheds light on cohorters’ concern for social recognition of the cohort in the Swiss population and the burden of clinical labor in such a cohort. The three situations account for difficulty in shifting epidemiology toward the technoscientific approach of the exposome in a country characterized by the predominance of biomedicine over public health approaches. While a form of technoscientific holism underlies the whole project and shapes many of the choices made in the present, in practice the analysis also makes clear that this scientific ideal is far from achieved and that public health utility, as well as population concerns, are also taken into account. In fact, these situations expose attempts to improve the social integration of the study into the Swiss scientific, political and social landscape.

Reduction and reductionism have various meanings, whether in painting, surgery, philosophy, or geometry. Common to all these definitions is the point that reductionism is an operation consisting of translating a whole, an entity, an object, a phenomenon, into something that retains some of its initial characteristics, but also implies a loss of quality, a shrinking, as its negative connotation makes clear. In the context of the exposome, it refers to the reduction of social conditions determining health to differences observed at the molecular level, and to the individualist and causalist explanatory framework of disease origins (Giroux, 2023). Reductionism is inherent to scientific practice. In social sciences and qualitative research, we also reduce the complexity of the realities we observe. We select certain elements over others, as I have for this paper. From this analysis it follows that reducing also involves a condensation, a keeping of what are considered the essential characteristics of the original. I suggest looking at situations of reductionism as moments of friction, trade-offs, and negotiation between different rationales and values, but also as moments of condensation which expose what is important in a specific context. I argue that these situations of reductionism can be understood as exposures of the “research environment” (Pinel, 2022) and exposures of what these scientists “care for” (Penkler, 2022). In the Swiss context of public health research, these situations of reductionism expose what makes the science of the exposome, its material, economic, and methodological constraints, as well as its imaginaries and values.

Rather than opposing holism and reductionism, I would insist on their indissociability in this conclusion, as they constitute two sides of the same coin. The situations I have described here are moments where I felt both enthusiasm and sympathy for what cohorters were aiming to build, and disappointment at the depoliticizing shrinking of the social environment I observed. These situations of reductionism are productive in the sense that they expose what cohorters work toward and care for in the present, and in the long term. They are productive in the present, not in actualizing an exposomic understanding of health, which is postponed to the future, but in bringing together various actors who are interested in public health and environmental health and are willing to place the question of environmental health on the government’s agenda. Working to build a national cohort and infrastructure that is scientifically solid can be interpreted as a way of reinforcing public health in a country characterized by the predominance of biomedical actors and institutions. Thus, this cohort can be viewed as a biomedicalization of public health research, as well as an attempt to socialize it through the broad category of the exposome, which leaves enough room to cover multiple understandings of the environment. Different scientific and political agendas can cohabit, but this also obscures their conflicting dimensions and political implications in terms of public health interventions over the long term. Those are postponed to an indefinite future, with the risk of neglecting the problematic issues of the present (Hoeyer, 2019). In the present, situations of reductionism expose rather the intricacies of the research environment in public health and cohorters’ work for the cohort’s scientific and social integration. Finally, insisting on the indissociability of holism and reductionism in exposomic research brings attention to what was left out but was nevertheless there (Jerak-Zuiderent, 2015). Some holistic forms of the “social” cannot become, in the causalist, determinist, biological regime of proof prevailing in public health research, in the imperative for data standardization and harmonization, in the pressure to publish from the “entrepreneurial university” (Pinel, 2022), in the challenges of recruiting the population. Reductionism is thus not only enacted in multiple ways, but is also a way of exposing the conditions of possibility for some versions of the social environment to become.

## Data availability statement

The datasets presented in this article are not readily available because ethnographic data collected in this study are not sharable. Requests to access the datasets should be directed to nolwenn.buhler@unil.ch.

## Ethics statement

The studies involving human participants were reviewed and approved by CER-VD Req-2019-01305. Written informed consent for participation was not required for this study in accordance with the national legislation and the institutional requirements. Informed oral consent was obtained at the beginning of each interview.

## Author contributions

The author confirms being the sole contributor of this work and has approved it for publication.

## Footnotes

^1^The term refers both to a concept and a subsequent measurement tool, put into use to identify the cumulative physiological impacts of environmental stressors on human health, and tentatively help explain the biological pathways by which social conditions are embodied. The concept is operationalized into a composite score assembling a changing set of biomarkers, which is then correlated with various established measures of social deprivation (Serviant-Fine et al., 2023).

^2^Operational meetings reunite the cohorters in charge of elaborating and implementing the technical procedures and work instructions necessary to build the cohort infrastructure, that is, all the steps necessary to recruit participants, obtain ethical approval, collect data and samples, establish data flows and biobanks.

^3^Switzerland is a federal country composed of 26 cantons, the federated states. Each has its own constitution, parliament, and government.

^4^Environmental concerns were only one of the motivations to participate in this cohort (see Bühler et al., 2023).

^5^The Swiss health system is highly biomedicalized, individualized, and privatized. It has been described as “highly complex, combining aspects of managed competition and corporatism (the integration of interest groups in the policy process) in a decentralized regulatory framework shaped by the influences of direct democracy” (De Pietro et al., 2015). Health insurance schemes reimburse mostly medical treatments and not prevention and health promotion programs. Those are also mostly geared toward individual responsibility, rather than promoting population or structural measures. Thieme et al. (2022) also show how processes of economic rationalization, bureaucratization, and digitalization frame the Swiss healthcare system.

^6^Roundup is the name of a herbicide containing glyphosate, initially commercialized by the firm Monsanto, then purchased by Bayer, a German pharmaceutical and agrochemical firm.

^7^This interview was done at the beginning of the study. The study results show that cohortees’ urine levels of glyphosate are far from any threshold of concern.
